# Differential Effect of Dopamine D4 Receptor Activation on Low-Frequency Oscillations in the Prefrontal Cortex and Hippocampus May Bias the Bidirectional Prefrontal–Hippocampal Coupling

**DOI:** 10.3390/ijms231911705

**Published:** 2022-10-03

**Authors:** Carolina Wilnerzon Thörn, Vasilios Kafetzopoulos, Bernat Kocsis

**Affiliations:** 1 Department Psychiatry at BIDMC, Harvard Medical School, Boston, MA 02215, USA; 2 Department of Psychiatry, Medical School, University of Ioannina, 45110 Ioannina, Greece

**Keywords:** schizophrenia, cognitive deficits, animal models, prefrontal cortex, hippocampus, oscillatory coupling, narrow-band delta oscillation, theta rhythm

## Abstract

Dopamine D4 receptor (D4R) mechanisms are implicated in psychiatric diseases characterized by cognitive deficits, including schizophrenia, ADHD, and autism. The cellular mechanisms are poorly understood, but impaired neuronal synchronization in cortical networks was proposed to contribute to these deficits. In animal experiments, D4R activation was shown to generate aberrant increased gamma oscillations and to reduce performance on cognitive tasks requiring functional prefrontal cortex (PFC) and hippocampus (HPC) networks. While fast oscillations in the gamma range are important for local synchronization within neuronal ensembles, long-range synchronization between distant structures is achieved by slow rhythms in the delta, theta, alpha ranges. The characteristics of slow oscillations vary between structures during cognitive tasks. HPC activity is dominated by theta rhythm, whereas PFC generates unique oscillations in the 2–4 Hz range. In order to investigate the role of D4R on slow rhythms, cortical activity was recorded in rats under urethane anesthesia in which slow oscillations can be elicited in a controlled manner without behavioral confounds, by electrical stimulation of the brainstem reticular formation. The local field potential segments during stimulations were extracted and subjected to fast Fourier transform to obtain power density spectra. The selective D4R agonist A-412997 (5 and 10 mg/kg) and antagonists L-745870 (5 and 10 mg/kg) were injected systemically and the peak power in the two frequency ranges were compared before and after the injection. We found that D4R compounds significantly changed the activity of both HPC and PFC, but the direction of the effect was opposite in the two structures. D4R agonist enhanced PFC slow rhythm (delta, 2–4 Hz) and suppressed HPC theta, whereas the antagonist had an opposite effect. Analogous changes of the two slow rhythms were also found in the thalamic nucleus reuniens, which has connections to both forebrain structures. Slow oscillations play a key role in interregional cortical coupling; delta and theta oscillations were shown in particular, to entrain neuronal firing and to modulate gamma activity in interconnected forebrain structures with a relative HPC theta dominance over PFC. Thus, the results of this study indicate that D4R activation may introduce an abnormal bias in the bidirectional PFC–HPC coupling which can be reversed by D4R antagonists.

## 1. Introduction

The concept of “animal models” of psychiatric diseases has undergone major clarifications in the past decades as models aimed at recapitulating a human psychiatric disorder and verified on the basis of face or predictive validity are often of unclear relevance to the etiology of the human disease. The currently used research framework for investigating mental disorders, formulated as Research Domain Criteria (RDoC) [1], identifies the role of animal research as clarifying the mechanisms, either genetic, molecular, physiologic, or behavioral, that lead to anomalies in specific domains relevant for understanding the origin of psychiatric diseases. Following this concept, this study focused on rhythmic synchronization of neural activity in higher order brain networks, essential in one of the main RDoC domains, cognitive function. Rhythmic coupling is a powerful, ubiquitous mechanism of functional coordination in local circuits and between different cortical networks; it plays key roles in numerous cognitive processes. Abnormalities in brain oscillations are well established in various psychiatric diseases, including schizophrenia, major depression, ADHD, and others, and were linked to cognitive dysfunction, characteristic for these conditions.

Cognitive dysfunctions represent a primary target in schizophrenia research and treatment. Although several decades of antipsychotics have brought a remarkable progress in limiting the impact of positive symptoms, these compounds proved unable to cure schizophrenia, indicating that the core deficits remained unaffected. Among these impairments are various cognitive deficits and negative symptoms, which, relative to positive symptoms, show better correlation with clinical prognosis [2]. Recognizing this fact, the focus, both in human studies and animal models, has been shifting to dysfunctional cortical microcircuitry. In particular, NMDA receptor pathology and developmental abnormalities of parvalbumin positive (PV+) GABAergic interneurons, consistently observed in human post-mortem studies [3,4] and in most animal models [5], raise the possibility that impaired network oscillations play a primary role in mediating cognitive deficits and pathology. In some human imaging studies for example, an intermediate oscillatory phenotype was shown to be a more fruitful correlation target than behavioral measures for identifying genetic biomarkers [6]. Extensive research during the past several years, demonstrating abnormalities in gamma (40–70 Hz) oscillations in human schizophrenia [7,8,9,10] and in animal models [11,12,13,14,15,16,17,18] lead to the proposal that a “gamma oscillatory endophenotype” underlies downstream phenotypic cognitive deficits of schizophrenia and even specific therapeutic targeting of oscillatory deficits has been suggested [19].

Although high frequency oscillations have been the main focus of ongoing schizophrenia research, low frequency synchronization is also impaired by the disorder, and this impairment is significantly understudied. These low frequency oscillations also depend on NMDA-R mechanisms and PV+ interneurons, and thus the cellular substrate of pathological alterations of fast and slow oscillations significantly overlap. Slow oscillations are behavior and region-specific; theta rhythm (5–10 Hz) represents an active state of the hippocampus (HPC) [20], while the dominant slow rhythm in the prefrontal cortex (PFC) is in the delta (2–5 Hz) range, active in task-related activities related to working memory [21]. Theta and wake-delta can also be recorded in different cortical areas where they appear related to specific tasks and serve as frequency-tagged communication channels for HPC and PFC, respectively [22]. The neural mechanisms involved, can also be investigated under urethane anesthesia, i.e., excluding behavioral confounds, where these oscillations can be elicited by electrical stimulation of the ascending arousal system in the brainstem reticular formation (nucleus reticularis pontis oralis–RPO).

The dopamine 4 receptor (D4R) was found abundantly expressed in PV+ interneurons as wells as in pyramidal cells in PFC and HPC [23,24,25,26,27]. Dopaminergic neurons in the VTA were shown to synchronize with substantial fractions of these cells at both delta and theta frequencies and gamma rhythm in the VTA was also modulated by delta rhythm in VTA and PFC and by the phase-coupled theta in HPC [21]. D4R agonists in high doses elicit aberrant gamma oscillations [28] comparable with that reported after administration of ketamine or other NMDA-R agonists [29]. Interestingly, ketamine’s action on gamma oscillation is at least partially depended on D4R in the thalamus but not in the PFC [29].

D4R mechanisms are essential for normal processes of attention [30] and there is evidence for its involvement in the pathology of cognitive decline in schizophrenia. Most importantly, clozapine, shown more effective than other antipsychotics against negative and cognitive impairments in schizophrenia [31,32], has a high affinity to D4R [33,34], in contrast to other antipsychotic drugs mainly targeting D2R. Thus, in this study we used the highly selective D4R agonist (A-412997) and antagonist (L-745870) to investigate the role of D4R mechanisms in low frequency oscillations potentially relevant to schizophrenia. We found that D4R activation led to a significant increase in wake-delta power in PFC and a significant decrease in HPC theta power indicating an abnormal shift in the communication between the PFC and the HPC, whereas L-745870 induced an opposite effect.

## 2. Results

### 2.1. Effect of RPO Stimulation on Forebrain Oscillations

**Figure 1:**
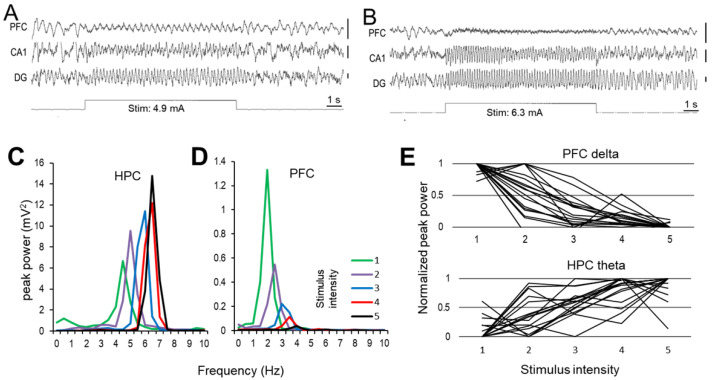
Hippocampus (HPC) and prefrontal cortex (PFC) oscillations induced by nucleus reticularis pontis oralis (RPO) stimulation in urethane anesthetized rats. (**A**,**B**) Sample recordings of PFC and HPC activity in control conditions in response to RPO stimulation (see marker channel) at low ((**A**) Low stimulation) and high intensity ((**B**) High stimulation). Note delta oscillation in the PFC and theta oscillation in the HPC and their characteristic changes; frequency increases in both signals, whereas amplitudes change in opposite directions, i.e., larger theta and smaller delta, as RPO stimulation intensity is increased. (Calibration in A and B: 0.5 mV). (**C**,**D**). Power spectra from a representative experiment demonstrating theta and delta peaks in the HPC (**C**) and PFC (**D**), respectively, at five levels of stimulus intensity (1 is the lowest and 5 the highest). (**E**) Changes in peak amplitude of HPC theta and PFC delta oscillations at different levels of RPO stimulation intensity, in control conditions (pre-injection) in all experiments (*n* = 18) of this study.

Neural activity was recorded and analyzed in the PFC and HPC as well as in the nucleus reuniens of the thalamus (nRE), which has connections to both forebrain structures. As described previously [35,36], forebrain activity in rats under urethane anesthesia alternated between “active” and “passive” states characterized in this model by rhythmic vs. non-rhythmic local field potential (LFP) signals, respectively. In active states, the dominant frequency of synchronized rhythmic activity in HPC was in the theta range (5–10 Hz), whereas in the PFC it was in the delta band (2–5 Hz) [22]. Both patterns produced sharp peaks in the signals’ autospectra (see below) which was markedly different from the irregular LFPs with wide distribution of their spectral power in passive states. As well-documented in prior studies [35,37], these states alternated spontaneously in undisturbed conditions but active states could also be reliably induced by electrical stimulation of the RPO (Figure 1).

RPO stimulation induced theta rhythm in the HPC and low frequency oscillation in the PFC within the delta range, with their exact characteristics depending on the intensity of RPO stimulation [22]. Thus, progressively increasing stimulus intensity led to opposing changes in the amplitudes of HPC theta and PFC delta oscillations whereas increasing the frequency of both rhythms. Figure 1 shows an example of irregular activity switching to stable delta rhythm in the PFC and theta in the HPC induced by short episodes (10 s) of RPO stimulation at low intensity (Figure 1A) and their characteristic changes after increasing stimulation intensity (Figure 1B). RPO was stimulated at five different levels of intensity, evenly distributed between the threshold and maximum, identified in each experiment as the lowest intensity eliciting HPC theta in most (>50%) trials and the highest intensity, after which theta frequency no longer increased. The stimulations then repeated five times at each level, randomized, within a ~30 min period, before, and immediately after drug administration, and again ~30 min later. As shown in the example (Figure 1C,D), in control conditions, the frequency of both signals increased within their characteristics theta and delta bands (4.9 to 6.8 and 2.4 to 4.4 Hz, respectively in this experiment), with increasing intensity of RPO stimulation, whereas their peak power changed in opposite directions, i.e., HPC theta increased and PFC delta decreased, in parallel. This trend between low and high stimulus intensities was present in all rats with no exception although the step-by-step changes were not necessarily linear in all experiment and varied with spontaneously alternating active and passive states at the time of stimulation [22] (Figure 1E). HPC theta increase and PFC delta decrease were significant (F_(4,50)_ = 18.975, *p* < 0.001, F_(4,50)_ = 15.68, *p* < 0.001, respectively), as were the increase in frequencies, as well (theta: F_(4,50)_ = 7.102, *p* < 0.001, delta: F_(4,50)_ = 29.133, *p* < 0.001).

### 2.2. Effect of Dopamine D4-Receptor Activation on Forebrain Network Oscillations

**Figure 2:**
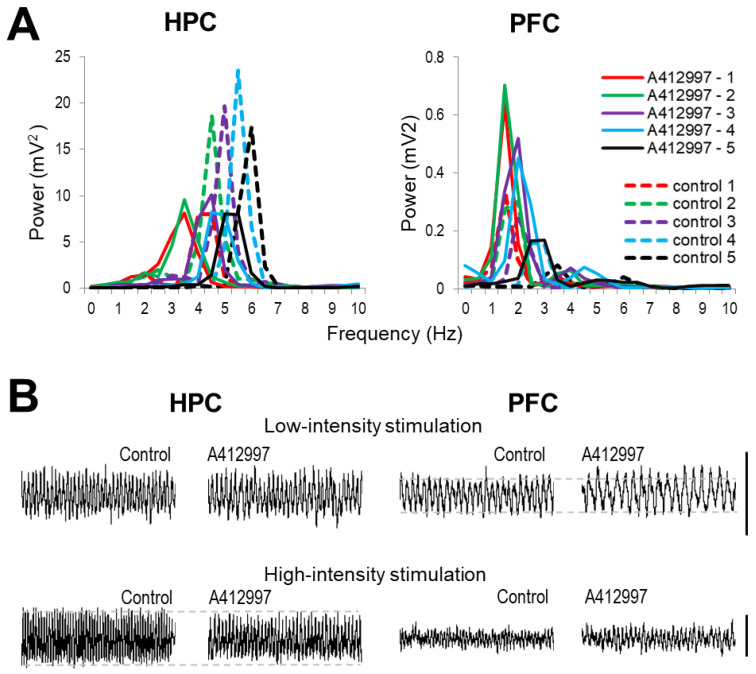
Changes in power spectra of local field potentials due to dopamine D4 receptor (D4R) activation in a representative experiment. (**A**) Power spectra of hippocampus (HPC) and prefrontal cortex (PFC) signals during nucleus reticularis pontis oralis (RPO) stimulation at different intensities before drug administration (control, *dashed lines*) and after injection of A-412997 (10 mg/kg) (*continuous lines*). Note consistent decrease in HPC theta and increase in PFC delta peaks at each level of stimulation. (**B**) Sample recordings (8 s segments) of HPC and PFC activity during low and high intensity RPO stimulation before drug administration (Control) and after injection of A-412997. (*Dashed lines* added to aid comparisons of pre- and post-injection signals at stimulations inducing strongest oscillations, i.e., low intensity for PFC-delta and high intensity for HPC-theta. Calibration in B: 0.5 mV).

After injection of the selective D4R agonist A-412997 (10 mg/kg), PFC delta increased while HPC theta decreased in amplitude and appeared slower. As shown in a representative example (Figure 2), theta peaks in the HPC autospectra were lower at all stimulation levels and delta rhythm in the PFC changed in the opposite direction. Note for example, that at high stimulation (level 5, black in Figure 2A and bottom-right sample trace in Figure 2B) PFC delta, generally diminished compared with low-intensity stimulation, was still present after injection whereas it was abolished in pre-injection control.

**Figure 3:**
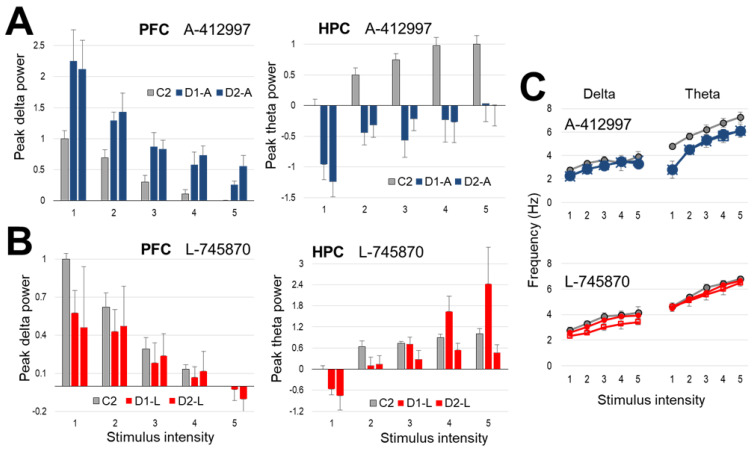
The effect of dopamine D4 receptor (D4R) agonist and antagonist on low-frequency oscillations in forebrain networks induced by nucleus reticularis pontis oralis (RPO) stimulation. (**A**,**B**) Peak delta power in prefrontal cortex (PFC) and theta in hippocampus (HPC) (mean ± SEM, normalized to the maximal response in pre-injection control, i.e., at stimulus level 1 for PFC and 5 for HPC) after injection of the D4R agonist A-412997 (*n* = 7) (**A**) and the antagonist L-745870 (*n* = 6) (**B**). RPO was stimulated at 5 different levels of intensity, repeated five times each within a ~30 min period, before (*C2—grey*), and immediately after drug administration (*D1-A—blue*, *D1-L—red*), and again ~30 min later (D2-A, *D2-L*). (**C**) Delta and theta frequency induced by RPO stimulation pre- (*grey*) and post-injection (*blue* and *red*) in the same experiments.

The changes in spectral peaks were quantified in a group of *n* = 7 rats, normalized in each experiment, and expressed relative to the highest power peak (i.e., at level 1 stimulation for PFC delta, and 5 for HPC theta) induced in control conditions (=1, see in Figure 3A). On group average, stimulation at this level increased the delta peak more than two times relative to the control, which remained stable for two consecutive series of stimulations (~30 min each), one after injection and one ~30 min later. As in the control conditions, peak delta power progressively decreased with increasing stimulus intensity post-injection as well, but it was higher than pre-injection at each intensity including even a change in threshold (see level 5). HPC theta was drastically reduced, even at the strongest stimulation level (Figure 3A).

Altered oscillations at different stimulus intensities were statistically analyzed using normalized theta and delta peaks in the autospectra. In the PFC, there was a significant increase (F_(1,69)_ = 11.602, *p* < 0.001) in peak power of the delta component compared with pre-injection control. There was also a significant increase in the dominant delta frequency (F_(1,69)_ = 16.557, *p* < 0.001, Figure 3C). In the HPC, there was a significant decrease (F_(1,69)_ = 8.51, *p* = 0.005) in the theta power compared to the control at each level of increasing stimulus intensity and the theta frequency were also significantly altered as it decreased for each level of increasing stimulus intensity (F_(1,69)_ = 5.028, *p* = 0.001).

**Figure 4:**
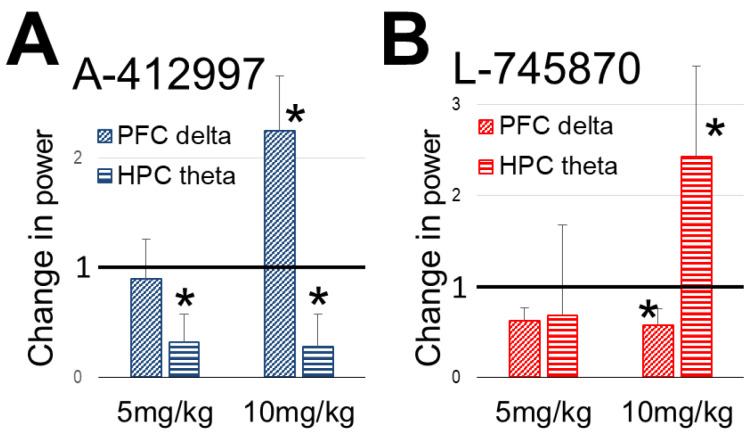
Dose–response effect of dopamine D4 receptor (D4R) agonist A-412997 (**A**) and antagonist L-745870 (**B**) on oscillatory activity, injected in 5 and 10 mg/kg doses. Change in peak power (mean ± SEM) at stimulus intensity, most effective in control condition (i.e., level 1 for prefrontal cortex (PFC) delta and level 5 for hippocampus (HPC) theta), expressed relative to control (=1, not shown) in each group. * indicates statistical significance (*p* < 0.05). Note that 5 mg/kg dose was administrated in smaller groups (*n* = 3 for agonist and *n* = 2 for antagonist) than 10 mg/kg (*n* = 7 and *n* = 6).

In three rats, A-412997 was injected in a lower dose (5 mg/kg) to compare with the effect of the higher dose, at the most effective level of stimulus intensity (Figure 4A). There was no significant change in PFC delta power after drug administration at a concentration of 5 mg/kg (F_(1,50)_ = 0.774, *p* = *ns*), in contrast to the 10 mg/kg dose (F_(1,50)_ = 16.557, *p* < 0.001). However, 5 mg/kg A-412997 was effective in the HPC; there was a significant decrease in theta power for both the 10 mg/kg and 5 mg/kg concentrations (F_(1,50)_ = 18.975, *p* < 0.001; F_(1,50)_ = 64.044, *p* < 0.001, respectively), compared to controls (Figure 4).

**Figure 5:**
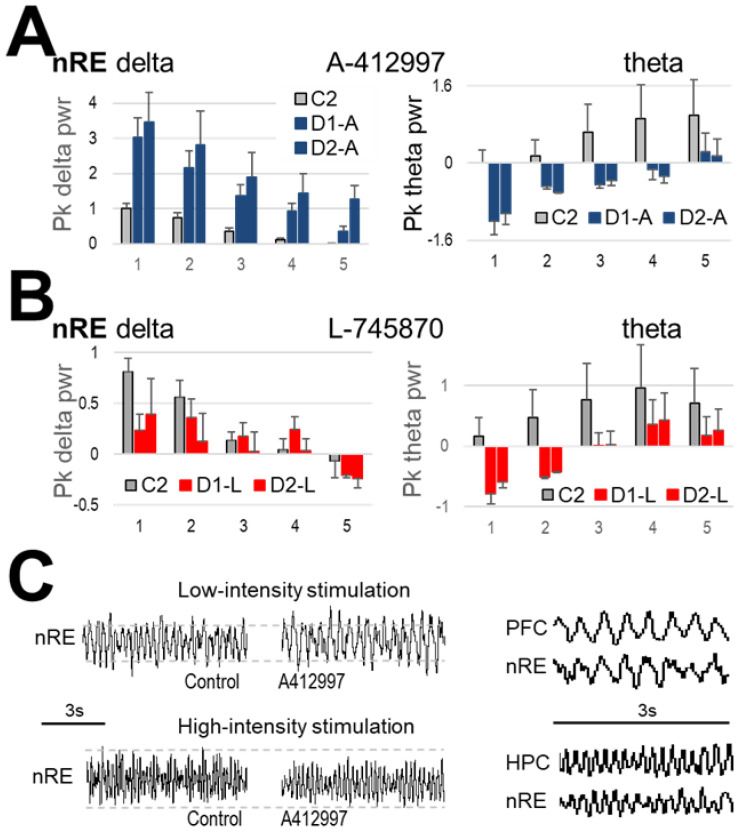
The effect of dopamine D4 receptor (D4R) agonist and antagonist on low-frequency oscillations in nucleus reuniens (nRE), induced by nucleus reticularis pontis oralis (RPO) stimulation. (**A**,**B**) Peak delta and theta power (mean ± SEM, normalized to the maximal response in pre-injection control, i.e., at stimulus level 1 for delta and 5 for theta peak power) after injection of the D4R agonist A-412997 (*n* = 7) (**A**) and the antagonist L-745870 (*n* = 6) (**B**) RPO was stimulated at 5 different levels of intensity, repeated five times each within a ~30 min period, before (*C2—grey*), and immediately after drug administration (*D1-A—blue*, *D1-L—red*), and again ~30 min later (D2-A, *D2-L*). (**C**) Sample recordings of nRE activity (8 s segments, simultaneously recorded with prefrontal cortex (PFC) and hippocampus (HPC) shown in Figure 3) during low and high intensity RPO stimulation before drug administration (Control, *left*) and after injection of A-412997 (*middle*). *Right*: comparison of delta waves in PFC and nRE (*top*) and theta rhythm in HPC and nRE (*bottom*), on an extended time scale (3 s segments).

In nRE, a bidirectional interconnecting node between HPC and PFC in the thalamus, strong theta and delta peaks were present in the autospectra, respectively largest at level 5 and 1 of stimulus intensity, and progressively declining as the stimulation changed (Figure 5). The effect of the D4R agonist was also in-line with that in the corresponding forebrain structures (Figure 5A); i.e., theta power decreased in the nRE after 10 mg/kg and 5 mg/kg (F_(1,50)_ = 14.018, *p* < 0.001 and F_(1,20)_ = 11.716, *p* = 0.003, respectively) whereas delta power increased at 10 mg/kg (F_(1,50)_ = 26.386, *p* < 0.001) but not at 5 mg/kg (F_(1,20)_ = 1.2081, *p* = 0.338), similarly to PFC and HPC, respectively.

### 2.3. Effect of Dopamine D4-Receptor Blockade on Forebrain Network Oscillations

The selective D4R antagonist L-745870 (injected in *n* = 6 rats, 10 mg/kg) elicited strong effect which was opposite to that after D4R activation (Figure 3B,C). There was a significant decrease (F_(1,50)_ = 4.078, *p* = 0.032) in peak PFC delta power and a significant increase (F_(1,50)_ = 3.89, *p* = 0.045) in theta peak power in the HPC (Figure 3B). More specifically, the increase in theta power was only observed on the more ventral HPC electrode in the dentate gyrus (DG) producing stronger theta LFP (See Methods). The antagonist did not affect delta peak power in the HPC (F_(1,50)_ = 0.384, *p* = ns) or theta power in PFC (F_(1,50)_ = 1.709, *p* = 0.197). L-745870 injected in 5 mg/kg dose did not appear to alter either rhythm (Figure 4B). However, the lower dose was only used in a pilot (*n* = 2) which does not allow to draw firm conclusions, supported by statistical analysis.

The increase in theta power was also observed in nRE after administration of D4R antagonist (F_(1,50)_ = 7.174, *p* = 0.001) (Figure 5B). The antagonist however did not affect delta peak power in the nRE (F_(1,50)_ = 1.036, *p* = 0.31), except during stimulation at level 1 (significant by a narrow margin; t_(5)_ = 2.036, *p* = 0.048) which elicits the highest delta power in control conditions.

## 3. Discussion

The aim of this project was to study the effects of the dopamine D4R interaction on low frequency oscillations in the PFC and the HPC. The results show that D4R activation suppresses theta oscillations in the HPC and enhances regular oscillations in the delta range in the PFC. D4R inhibition had the opposite effect; it decreased delta power in the PFC and increased theta power in the HPC. In a previous study [12], D4R activation using the same compound administered in the same dose was shown to elicit abnormal gamma enhancement in freely moving rats.

For this study we chose anesthetized over freely moving rats to avoid behavioral confounds strongly affecting forebrain oscillations. Urethane was preferred to other anesthetics as it induces long lasting anesthesia and allows low-frequency oscillations generated in the rats, even though gamma oscillations are suppressed in this model. This model has been used for decades in a wide range of applications to study network mechanisms of low-frequency rhythms in different systems [38,39,40,41,42,43,44]. Specifically, narrow-band regular delta (2–5 Hz) oscillations in the PFC occur in this preparation [22,45] where theta rhythm has been routinely recorded in HPC [35], spontaneously alternating with wide-band delta (large irregular activity [40]) in both structures. It was consistently demonstrated that stimulation of RPO, a structure in the brainstem reticular formation essential for controlling the arousal level or awakening the brain, induces these patterns of activity under urethane anesthesia [22,44,46,47], which in freely moving animals specifically appear during cognitive tasks [20,21]. It should be noted that the delta-range activity elicited by RPO stimulation generated narrow sharp peak in PFC autospectra, i.e., similar in its character to the co-occurring HPC theta peaks and different from wide-band delta activity in passive states which serves as model for sleep EEG in unanesthetized animals. Using this model was also justified by the primary focus of this study to analyze synchronization at lower frequencies detached from fast, e.g., gamma, rhythms. Freely moving animals receive inputs from a number of sources, such as visual and audio sensory inputs and from sources related to motor activity, which generate higher frequency oscillations that interfere with slower rhythms. Urethane anesthesia allows studying delta-theta rhythms in a reduced preparation excluding the effect of these extra inputs.

There have been many studies conducted on gamma (40–70 Hz) oscillations and its relation to schizophrenia-related and cognitive impairments [13,24,48,49,50], but few studies have focused on slower rhythms and their involvement in cognitive functionality. Advances in studying gamma synchronization in the past decade led to the concept of “gamma oscillatory endophenotype” [19,51]. Gamma oscillations are part of an oscillatory hierarchy however; their function and especially their coordination between distant networks require state and task-dependent low-frequency modulation [52,53,54,55]. Locally generated gamma oscillations are dependent on low-frequency oscillations to synchronize neural activities in distant regions. Delta or theta oscillations and their gamma-modulation is region specific. HPC theta is well studied, including its role in synchronizing HPC with PFC circuits [56]. The role of delta drive originating in PFC and reaching HPC via the nRE [57] providing another channel of PFC–HPC communication in the other direction [22] is less understood. Abnormal gamma oscillation in animal models was also shown to be modulated either by theta or delta rhythms depending on the specific receptor targeted by the psychoactive compound to induce schizophrenia-like behavior [52]. The literature on slow rhythms in human schizophrenia remains equivocal but it was suggested [58] that the balance between the PFC and HC pathways coordinating their communication is biased in schizophrenia away from theta towards delta coordination.

The two types of slow oscillations play different roles in cognitive function. A study on cognitive related tasks in normal healthy humans [59] also characterized the information flow between the PFC and the HPC as being bidirectional, where the drive from the HPC to the PFC is found to be more dominant over the drive from the PFC to the HPC. This suggests that the HPC theta is more dominant over the awake delta in the PFC. This phenomenon was also observed in the in vivo animal study [21], where rats placed in mazes performed a cognitive task in which they eventually had to make a decision between turning to the left and turning to the right. Theta was found to participate from the beginning of the task until decision making, while a regular 4 Hz oscillation (i.e., in delta range) was present solely at the point of the decision making. These studies point to different functional roles of slow oscillations in the theta and delta frequency bands, in both human and animals. The results obtained in our project indicate that D4R mechanisms might affect this dynamics, as opposite effects on the PFC and HPC oscillations were observed following D4R activation and inhibition.

The exact mechanism of such an opposite D4R-dependent effect on the PFC and HPC remains to be explored in future studies for better interpretation of these results. It is not clear for example, which type of neurons are being preferentially affected in each specific region. D4Rs are present in both inhibitory interneurons and excitatory pyramidal cells [23,24,25,26,27], both essential components of network oscillations. D4Rs is considered to be a D2-type receptor coupled to the G protein G_i_ with an overall inhibitory effect on neuronal activity; as the activated receptor inhibits the adenylate cyclase, thereby reducing the concentration of the second messenger cyclic AMP. This might suggest for example that the neurons being affected in the HPC are pyramidal cells whereas the neurons affected in the PFC are interneurons. Further studies are required to determine specifically and with certainty which neurons are being affected in the PFC and HPC, respectively.

D4R agonist was systemically injected in two different concentrations and while it had an effect in the HPC in both concentrations, only the injection of the high dose resulted in a significant change in the PFC. This indicates a dose-dependent effect (in a relatively small sample) on delta oscillation in the PFC and a significant effect of the D4R agonist on theta in the HPC even at the 5 mg/kg dose. Dose-dependent effect of D4R agonism on network oscillations was also reported in freely moving rats in a previous study [12], where injection of A-412997 increased gamma activity in the PFC in a dose-dependent manner. The strength and character of gamma activity induced by the high dose (10 mg/kg) was similar to the aberrant gamma enhancement in rodent models of schizophrenia elicited by NMDA-blockade [13,51,60,61,62,63]. The doses in the current study were chosen to investigate potential changes in slow oscillations, which might occur in parallel to such aberrant gamma enhancement. Note, that gamma oscillations under urethane are grossly suppressed but slow rhythms can be elicited in a controlled manner. These slow rhythms in freely moving rats are, in turn, dependent on behavior and are thus more difficult to handle.

D4Rs play an important role in normal cognitive functions, such as attention and memory [30], and several studies have shown that D4R agonists increased cognitive performance in low doses [64,65,66] but lead to severe impairment after the injection of higher doses [66]. At this concentration it could provide a high level of background D4R activation upon which the effect of endogenous dopamine would be washed out, resulting in impaired performance, and perhaps even schizophrenia-like cognitive deficits. Changes in network oscillations, i.e., abnormal gamma activation [13] and disturbing the delta-theta balance in PFC–HPC communication shown here, may be important components of the neural mechanisms leading to this effect.

HPC is directly connected to PFC, but PFC is connected reciprocally with HPC through nRE, a nucleus residing in the midline thalamus [67,68]. nRE was shown to participate in multiple functions that also implicate both PFC and HPC, such as attention, spatial and affective memory, and stress [69,70,71,72,73]. HPC to PFC direct communication is synchronized by theta rhythm, while PFC to HPC communication, mediated by nRE, occurs through delta rhythmic synchronization [22,74,75]. D4 agonist altered delta power in nRE, as it did in PFC. nRE and PFC delta communication is thought to enable PFC in synchronizing activities in other brain regions. In contrast, D4R agonism disrupted theta power in nRE, possibly contributing in aberrant theta propagation from HPC to other structures, including PFC. The nRE may relay PFC–HPC communication facilitated by rhythmic synchrony, and thus phase modulation of gamma activity in these structures, either at delta or theta frequencies and the reactivity of nRE in both delta and theta bands after D4R activation further supports this notion.

The D4R antagonist was found to have an effect of its own when injected by itself. This could be due to one of two factors. First blocking dopamine that might already be present in the rat brain during anesthesia from interacting with the receptor, i.e., antagonizing the activation of D4Rs. The second factor could be due to the D4R having an intrinsic effect of its own [76], suppressing the spontaneous receptor signaling, i.e., acting as an inverse agonist. Thus, it might reduce or moderate the consequences of potential overactivation of D4Rs and improve cognitive performance in pathological situations. The selective antagonist L-745870 was previously reported [77] to be inefficient as an antipsychotic in schizophrenia in clinical trials. The results of this study suggest however, that a D4R antagonism could possibly represent an effective therapeutic target, not as an antipsychotic aimed for the treatment of the positive symptoms, but aimed for the treatment of the cognitive impairments. It should be noted however, that as previously reported [78], there are many genetic variations of the D4R in the human population that should be taken into account as it may provide a challenge in the future discovery of novel medications. Notably, the 7-repeat variant is linked to variations in gamma oscillations [79]. A highly specific new medication may not be the optimal solution as the inter-individuality between individuals with different genetic encodings for the D4R could result in different drug responses.

## 4. Materials and Methods

### 4.1. Animals

Male Sprague Dawley rats were used to perform this experiment. A total of 18 rats were used weighting between 350 and 500 g. All experiments were performed in accordance to the Beth Israel Deaconess Medical Center’s Institutional Animal Care and Use Committee (IACUC).

### 4.2. Drugs

The D4R agonist utilized was A-412997 from Tocris (Abcam Inc., Cambridge, US). A 50 mg amount of the A-412997 was dissolved in 5 mL saline and was used to prepare solutions with concentrations of 10 mg/kg and 5 mg/kg. A subcutaneous dose was injected in each rat in accordance to its weight. A-412997 is a highly selective D4R agonist with affinity at rat D4 and human D4.4 receptors (Ki values are 12.1 and 7.9 nM, respectively). It is a potent, full agonist at rat dopamine D4Rs (EC50 = 28.4 nM) and has no affinity for other dopamine receptors (Ki > 1 μM). It is able to rapidly cross the blood–brain barrier following systemic administration (see www.abcam.com/a-412997-dihydrochloride-d4-agonist-ab120581.html, our last access date: 1 November 2022).

The utilized D4R receptor antagonist was L-745870 from Tocris (Abcam Inc., Cambridge, US). A 50 mg amount of the L-745870 was dissolved in 2,5 mL saline and the concentrations of 10 mg/kg and 5 mg/kg were derived. L-745870 is a brain permeable highly potent and selective D4R antagonist (Ki values are 0.43, 2300 and 960 nM at D4, D3 and D2 receptors, respectively) (see www.abcam.com/a-412997-dihydrochloride-d4-agonist-ab120581.html, our last access date: 1 November 2022).

Urethane (ACROS Organic.™, Fisher Scientific) was used to induce anaesthesia and non-competitive NMDA-receptor antagonist ketamine (Ketaject, Phoenix™, St. Joseph, MO, USA) was utilized for euthanasia of the rat prior to decapitation.

### 4.3. Surgery

Surgery was performed under urethane anesthesia, which unlike other anesthetics, allows theta generation under controlled circumstances. The rat was injected intraperitoneally with two doses of urethane based on a concentration of 1 g/kg, with a one-hour interval. If full anesthesia was still not reached following the two urethane doses, a small dose of ketamine was injected (from 3.5 to 5 mg/kg). A total of six electrodes were implanted in the rat for recording in accordance to the stereotaxic coordinates, using the rat brain atlas of Paxinos and Watson [80]. Specifically, two single-wire electrodes were implanted in the right and left PFC (AP: +3.2, DV: −4.8, ML: +0.5 and −0.5, respectively) and two twisted double-wire electrodes implanted in the dorsal and ventral HPC (AP: −3.7, DV: −3.5, ML: +2.2 and AP: −6.0, DV: −6.2, ML: +5.7, respectively) and in the nRE (AP: −2.5, DV: −7.5, ML: 0.0)–the coordinates reflect the location of the longer electrode of the twisted pair whereas the shorter was located 0.8–1.2 mm dorsally. The electrodes were cemented to the bone using methyl methacrylate to secure them in place and then connected to the recording apparatus (A.M. Systems) for data collection. In addition, one pair of electrodes were placed in the RPO (AP: −8.0, DV: −8.0, ML: +1.5) through a guiding tube and connected to the stimulating apparatus (Master 8, A.M.P.I.)

### 4.4. Brain Cryosectioning and Histology

After the surgery and the data recording, the rats were euthanized with a 1 mL injection of ketamine and decapitated for the removal of the brain. The brain was stored at a low temperature in a 10% formalin solution (Fisher Scientific) in a glass vial. The formalin solution in the glass vial was exchanged for a 20% sucrose solution (Fisher Scientific), prior to sectioning. After removing the brain from the sucrose solution and freezing it with dry ice, a freezing microtome (Microm HM 450, Thermo Scientific) was used for slicing sections 50 μm thick. The brain slices were separated according to the rat brain atlas in a Petri dish containing PBS, and then mounted on Superfrost plus microscope slides using gelatin buffer [Gelatin type A (Acros), chromium(III) potassium sulfate (Fisher Science Education), Sodium Azide (Fisher Scientific)]. The slices were left to dry before being stained in Cresyl Violet (Fisher Scientific) staining. After drying from the staining, the slides were cover-slipped with Permaslip (Alban Scientific Inc.) for preservation and a light microscope was used for the analyses of the electrode placements.

### 4.5. RPO Stimulation and Data Recording

Electrophysiological recordings were collected using the DASYLab 7.0 software. LFPs were recorded via the implanted electrodes and lasted approximately three hours (197 ± 11 min). RPO was stimulated by 0.1 ms square waves, 100 Hz trains of 10 s duration, separated by 1 min, using a programmable stimulator (Master 8, A.M.P.I.) through flexible isolator unit to generate constant current pulses (Iso-Flex, A.M.P.I.) First, the range of RPO-stimulation intensity was established in each particular experiment. These parameters (similar to LFP recordings in deep structures) depend on various technical factors, including the exact location of the stimulation electrodes, the condition of the ascending pathway, etc. and thus have to be set individually in each rat before the start of the experimental protocol. Two parameters were identified, the minimum (i.e., the threshold) stimulus eliciting theta rhythm in the HPC in most (>50%) trials, and the maximal stimulus beyond which no further increase in theta frequency is observed. The effective stimulus intensity varied between 0.04 and 1.0 mA in individual experiments; the range (i.e., max-min) was 0.57 ± 0.04 mA. Thereafter repeated measurements of stimulation sequences at different intensity values within the two thresholds were performed. Each stimulation session consisted of 10 s-long stimulation epochs, 1 per minute, repeated in random order 5 times at each of the 5 intensity levels, thus completing a stimulation session lasting for 31 ± 2 min (range: 25–47 min). These sessions were executed in each rat in control condition immediately before each injection and then repeated starting 3–6 min after drug injection and again starting 41 ± 4 min post-injection. All signals along with stimulation markers on a separate channel were recorded continuously during the entire experiment. For analysis, 8 s-long segments during stimulations were selected off-line, ignoring the potential transient induced by turning on and off the stimulator.

### 4.6. Data and Statistical Analysis

DASYLab 7.0 and the MATLAB software, in addition to Microsoft Excel, were used to analyze the recorded EEG data. The 4 Hz and theta peak power values were extracted for each channel and stimulation intensity, and group averages were calculated from the normalized data (dependent variable) while the independent factor was either stimulus intensity to validate the technique (Section 2.1) or drug administration (pre- vs. post-injection, Section 2.2 and Section 2.3). LFP recordings were normalized to the maximal response in pre-injection control (set to =1, see Figure 3 and Figure 5), i.e., at stimulus level 1 for delta and 5 for theta peak powers. The standard error was calculated and one-way ANOVA was applied for the statistical data analysis. A *p*-value less than 0.05 was considered significant. To further analyze the differences on each specific level of stimulus intensity and drug effect the student’s paired t-test was applied with a Bonferroni correction.

## 5. Conclusions

The present data demonstrate the importance of the D4R mechanism for oscillatory synchrony and thus connectivity between the HPC and the PFC, vital for the cognitive functionality in the brain. Whereas the D4R activation resulted in an abnormal shift in the PFC–HPC communication, D4R inhibition had an opposite effect. These finding suggest that D4R antagonism may be a potential target for future pharmaceutical research aimed at the treatment of the cognitive impairments in schizophrenia.

## Data Availability

The data presented in this study are available on request from the corresponding author. Re-use and re-distribution of the experimental data will require prior approval of the corresponding author.
